# Professional conceptualisation and accomplishment of patient safety in mental healthcare: an ethnographic approach

**DOI:** 10.1186/1472-6963-11-100

**Published:** 2011-05-14

**Authors:** Jennifer Plumb, Joanne Travaglia, Peter Nugus, Jeffrey Braithwaite

**Affiliations:** 1 Centre for Clinical Governance Research, Australian Institute of Health Innovation, University of New South Wales, Level 1, AGSM Building, UNSW, Sydney NSW 2052, Australia

## Abstract

**Background:**

This study seeks to broaden current understandings of what patient safety means in mental healthcare and how it is accomplished. We propose a qualitative observational study of how safety is produced or not produced in the complex context of everyday professional mental health practice. Such an approach intentionally contrasts with much patient safety research which assumes that safety is achieved and improved through top-down policy directives. We seek instead to understand and articulate the connections and dynamic interactions between people, materials, and organisational, legal, moral, professional and historical safety imperatives as they come together at particular times and places to perform safe or unsafe practice. As such we advocate an understanding of patient safety 'from the ground up'.

**Methods/Design:**

The proposed project employs a six-phase data collection framework in two mental health settings: an inpatient unit and a community team. The first four phases comprise multiple modes of focussed, unobtrusive observation of professionals at work, to enable us to trace the conceptualisation and enactment of safety as revealed in dialogue and narrative, use of artefacts and space, bodily activity and patterns of movement, and in the accomplishment of specific work tasks. An interview phase and a social network analysis phase will subsequently be conducted to offer comparative perspectives on the observational data. This multi-modal and holistic approach to studying patient safety will complement existing research, which is dominated by instrumentalist approaches to discovering factors contributing to error, or developing interventions to prevent or manage adverse events.

**Discussion:**

This ethnographic research framework, informed by the principles of practice theories and in particular actor-network ideas, provides a tool to aid the understanding of patient safety in mental healthcare. The approach is novel in that it seeks to articulate an 'anatomy of patient safety' as it actually occurs, in terms of the networks of elements coalescing to enable the conceptual and material performance of safety in mental health settings. By looking at *how *patient safety happens or does not happen, this study will enable us to better understand how we might in future productively tackle its improvement.

## Background

### Patient safety and mental healthcare

Despite the rapid expansion of the patient safety literature over the last ten years, work on patient safety in mental healthcare 'has hardly begun' [[1]: xi]. Key patient safety texts [e.g. [2,3]] routinely fail to mention psychiatric or mental healthcare. It is unclear whether this is because the principles of patient safety are assumed to be equally applicable to mental health as to hospital-based medical and surgical care, or whether mental health is considered so different an environment as to require separate treatment. Either way, for the safety agenda to move forward, research into what patient safety means and entails in the mental health context is a fundamental requirement. This is the principal aim of this project.

The apparent disregard for mental health in the patient safety literature is mirrored by a neglect of 'patient safety' as a multi-dimensional concept in the mental health literature. Three distinct perspectives emerge from existing research into safety in mental health settings. The first, an operational perspective, is primarily focussed on description and guidance about discrete events and interventions, such as suicide prevention [e.g. [4]] and the anticipation and de-escalation of violent or aggressive behaviour [e.g. [5]]. The second, following Foucault [6], is a critical view of the role of mental health services as instruments of social control, preserving order (safety) in wider society by removing the 'disordered' (who are perceived as a risk or danger to the public).

Thirdly, there is a smaller body of work which examines safety in the context of everyday life and practice in mental health settings. These studies employ elements of the ethnographic tradition. Ethnographic methodology involves prolonged immersion of the researcher in the setting of interest in order to understand social phenomena from the 'inside' of the sites of their production. Many contemporary studies of this type [e.g. [7-9]] emphasise the central role of risk assessment and of 'keeping order' in structuring the conduct of mental healthcare, but generally do not focus on safety as their principal topic of interest. These studies also reveal that the patient is usually perceived as the principal locus of risk and its management, and that staff themselves, as well as the public at large, are considered to be 'at risk' if safety is not preserved. Such findings point to a distinctive conceptual framework surrounding risk and patient safety in mental health.

While each of the three perspectives contributes to our understanding of safety and mental health, they fall short of comprehensively articulating this conceptual framework and how it differs from prevailing notions of patient safety which are derived from the concerns of the medical and surgical sectors. This gap in knowledge is important if we are to avoid inappropriate safety interventions and improvement initiatives being implemented in the mental health context. There is also a lack of research illuminating what safe practice means to mental health professionals and how these meanings are enacted in everyday professional life. This is crucial in terms of the policy imperative to improve patient safety, because without such knowledge it is difficult to design improvement initiatives which chime with professionals' experience of trying to keep their services safe and the barriers they face to doing so. Intending to help fill this gap, the present project explores professional practice in mental healthcare as a nexus of social, historical, institutional and personal influences and tensions which come together in particular times and places to produce safe or unsafe practice.

### Ethnography and its importance for patient safety research

Qualitative, and particularly ethnographic, research approaches are increasingly recognised by patient safety experts [e.g. [10,11]] as offering a valuable contribution to the understanding of patient safety. They provide access to the contextual and cultural factors that contribute to the production of safety and error. This is because 'ethnographic observations can explore how the norms and rituals of professional practice can have potentially latent consequences for safety' [[12]: 164]. This type of research gives us a way of unpacking why improvement efforts often fail [13]. It equally offers the opportunity to examine how staff manage to keep healthcare environments and interventions safe most of the time (it is generally argued that around ten per cent of admissions to hospital are harmed by the healthcare they receive [14]). The longitudinal nature of ethnographic research allows for the observation of events and human interactions unfolding in real time. It offers an alternative perspective to the 'theoretical orthodoxy' of patient safety research, dominated by ideas derived from the application of systems science and human factors engineering in other industries [15,16]. Although these latter approaches have led to valuable improvements in safety, they are based on assumptions about safety derived from humans acting on (largely predictable) machine systems rather than on or with (much more unpredictable) humans. They cannot account for the particularities of healthcare, where contextual (social, cultural, personal, and interactional) factors play a fundamental role in what, and how, things are done.

There is now a growing realisation amongst some leaders of the patient safety movement that, in spite of concerted political, policy and research attention over the past decade, progress in reducing rates of iatrogenic harm has been disappointingly slow [17,18]. There is therefore a strong argument to be made for a return to 'first principles' in patient safety research, where prior assumptions about what safe practice is and how it is best achieved are discarded. Ethnographic approaches can enable us to build a picture of how safety is accomplished and understood at the front line of care, unencumbered by the assumptions of previous research. They can free us to examine instead the assumptions about safety which are embedded in professional practice and in the actions, interactions, discourses and materials which comprise it. We can also avoid a monolithic conceptualisation of patient safety by teasing out the role of multiple safety imperatives - historical, social, moral, legal, and institutional - in the conduct of clinical practice.

In support of these arguments, Øvretveit [[19]: 1782] states that as social scientists we must find ways to 'ensure room for both the currently much-in-demand instrumentalist research that provides practical solutions to problems, and for research which is critical and problematises current ways of thinking and acting. There is also a need for researchers who take the more critical stance to show how their research ultimately may be more practically useful as it may probe the issues more deeply.' Ethnographic research offers the opportunity to achieve this.

### Theoretical orientation

The theoretical and analytical framework shaping the project is derived from a body of social theory termed the 'practice approach' [20]. In the broadest terms this approach holds that social phenomena can be understood through examination of mundane, everyday human practices. The observation of everyday practices as they are carried out across time and in space offers rich opportunities for studying the social construction among professionals of concepts of safety and risk, as well as the actual accomplishment of safety, without resorting to attempts to 'get inside the minds' of participants. Notable empirical applications of this perspective using ethnographic methodology include examinations of the interactive production of safe practice on construction sites [21], and the practical accomplishment of cardiac telemonitoring [22].

Of particular interest to this study are those pragmatist theories which conceive of social phenomena as actively constituted, highly situated networks of practices, actions, ideas, artefacts and people. Such an approach is useful to our aim of returning to 'first principles' to articulate the building blocks of patient safety as they are revealed in professional practice. This approach is exemplified in actor-network theory (ANT) [e.g. [23]], later critical refinements of ANT [e.g. [24]], the notion of 'action nets' devised by Czarniawska [25] and the 'networks of practices' explored by Nicolini [22]. In a rare example of development of these ideas in relation to patient safety, Mesman [26] has described the accomplishment of sterility during central venous catheter insertion in a neonatal intensive care unit. She uses the notion of the 'safety net', attempting to articulate the 'fibres' of this net which, knotted together, enable sterility to be maintained. The fibres are the practices already in place, which usually recede into the background in research into safety - 'the elements that constitute the fabric of 'normal' practice' (1706). She emphasises the point that rather than focussing on missing parts of the net (when errors occur), it is vital to learn about the elements of the existing context and activity enabling the net to remain knotted together.

In this study the network metaphor will operate on multiple levels. Firstly it will be used conceptually, to enable us to articulate the networks of meanings contributing to different professionals' conceptualisations of safety. Secondly, the metaphor will be used as a methodological aid to help us trace the connections between people, artefacts, and organisational, legal, moral, professional and historical safety imperatives which come together to 'perform' safe or unsafe practice. Thirdly, the insights offered by identification and analysis of these networks of heterogeneous elements (humans, materials, ideas and practices) will be compared with the insights available through more conventional analyses of social networks or communities of practice (in which only humans are considered actors).

This study therefore seeks to use ethnographic methods, informed by the conceptual principles of actor-network and other practice-focussed theories, to illuminate what patient safety means in mental healthcare. Such an approach will enable us to articulate the concatenation of heterogeneous elements enabling its performance [[27]: 107].

### Research questions

These concerns are encapsulated in the research questions guiding the study.

1. How are mental health professionals' concepts of safety and risk constructed?

a. What is the nature of these understandings?

b. How do clinicians legitimise and sustain them?

2. How do these professionals accomplish safe practice?

a. Can we trace the network of connections between human, material, ideological, historical and institutional elements coalescing to produce safe or unsafe care?

b. Is the analysis of the discourse, narrative, activity, space and objects used in the course of professional practice a useful method for doing this?

3. What implications do these findings have for patient safety research and policy?

a. What do they infer for the way the patient safety movement defines safety problems and appropriate methods to tackle them?

b. What alternative approaches to improvement can be recommended as a result of this work?

## Methods/Design

### Type of study

**Figure 1:**
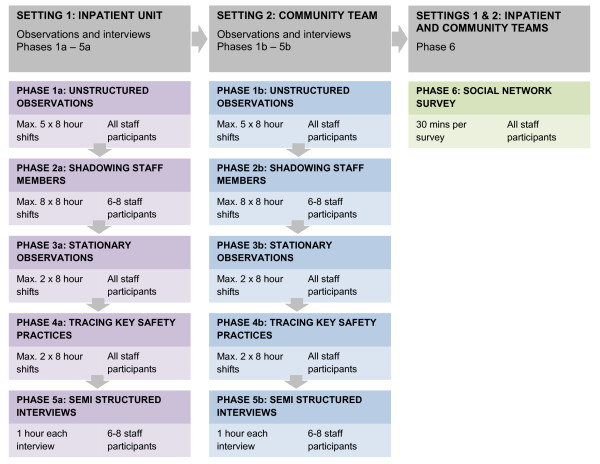
**Overview of study process**.

This is a qualitative study designed using an ethnographic methodological approach. An overview of the process is provided in Figure 1. Ethnography engages a variety of methods to build as detailed a picture as possible of the setting(s) under study, in an attempt to understand more about what the people in that setting 'experience as meaningful and important' [[28]: 2]. Ethnographers are sometimes described as data 'omnivores' [[29]: 18] because they use any sources of information about and from the sites of study that will help them reach such an understanding. Broadly based on social constructionist ideas, this approach does not assume that there is a single objective reality 'out there' waiting to be discovered and described, but rather seeks to 'reveal the multiple truths apparent in others' lives' [[28]: 3] and the ways in which these realities are constructed.

### Settings and participants

Two mental healthcare settings have been chosen for this study, and the six phases of data collection (see table 1 below) will be repeated in each. The first setting is an acute inpatient ward, and the second is a continuing care community service, both of which are located in the same hospital in New South Wales, Australia. The research participants are the approximately 50 mental health staff members working in these settings, comprising multi-disciplinary teams of doctors, nurses, psychologists, occupational therapists and social workers.

**Table 1:** Ethnographic data collection framework

PHASE	ACTIVITY	PURPOSE	LOGISTICS	PARTICIPANTS	OUTPUTS
**1.** Max. 5 × 8 hour shifts	Initial unstructured observations	• Familiarisation with space and use of space • Familiarisation with rhythm of setting • Starting to build relationships and trust • Determine who is amenable to shadowing • Identify key locations for stationary observations • Identify key policy/guideline documents	• Background observation of activity (potentially accompanying key initial contact in the setting) • Introductions with staff members • 5 shifts on different days of the week (over 2 weeks) (= max 40 hours)	All staff	• Map of settings • Timetable of key regular events • List of 6-8 key informants • Collected documents for later analysis (e.g. policies, guidelines) • Fieldnotes

**2.** Max. 8 × 8 hour shifts	Shadowing staff members	• Observe mechanics of interactional construction of safe practice • Observe interactions newcomers/old-timers • Observe use of tools/artefacts/environment • Identifying key practices to follow in later stage	• Shadow each staff member for 1 shift/part shift • Audio recording of key meetings attended by staff member • Field notes of informal talk • Field interviews	2 doctors 2 nurses 2 allied health 2 managers	• Map of practices of each key informant - how they construe patient safety and how they go about trying to maintain it • Fieldnotes • Transcriptions of meetings

**3.** Max. 2 × 8 hour shifts	Stationary observations in key locations	• Observe role of key artefacts in constitution of safety (e.g. phone in nurse's station; filing cabinet etc). • Observe patterns of movement of staff	• 2 locations, 1 shift each (max. 16 hours)	All staff	• Fieldnote account of how artefacts and space play a role in the constitution of safety • Actor-artefact network map

**4.** Max. 4 × 8 hour shifts	Tracing key practices	• Observe the unfolding of specific practices previously identified as key to preservation of safety • Observe differences in activity when practice is in the course of the everyday (e.g. admission/ discharge) and, if appropriate, when it follows breakdown in order (e.g. incident review)	• 1 'everyday' practice over the course of 2 shifts • 1 practice dealing with deviation from the normal (i.e. when safe practice has broken down in some way)	Staff involved in practices chosen	• Map of 'practice nets' involved in practices key to preservation of safety • How practice nets change when safety breaks down

**5.** Approx. 1 hr per interview	Interviews	• Elicit narrative accounts of safety preservation • Observe how the meaning of safety is constructed by different professionals - what 'rules and resources' do they draw on? • Test emerging findings/maps of practices	• 6-8 interviews - audio recordings	6-8 key informants from phase 2	• Transcripts for analysis

**6.** Approx. 30 mins per survey	•Social network survey	• Provide triangulation of observation and interview data • Map overall patterns of communication about safety issues within and between the two settings under study	• Administer a social network questionnaire to all staff in the inpatient team and community team under study.	All staff	• Social network diagrams providing visual representation of patterns of safety communication

This particular combination of settings was chosen for several reasons. Firstly, the two services have an overlapping client group; as clients experience acute episodes of illness, they may be admitted to the inpatient unit, and on discharge may return to or enter the care of the community team. This overlap will facilitate the examination of safety issues arising when care is transferred from one team to another, when a client leaves or is admitted to hospital. Secondly, the co-location of the two services will enable analysis of the levels and types interaction between their staff members on issues of safety, especially during the shadowing (phase 2) and social network analysis (phase 6) phases.

Most patient safety research in mental health has focussed on inpatient settings, and so a comparison of safety concerns and strategies between inpatient and community settings will be a valuable and novel contribution to the knowledge base. The difference in client acuity and in the purpose of care between the two settings (stabilisation of crisis versus long term case management of chronic but stable problems) will also provide a point of comparison for professional conceptualisation and enactment of patient safety. Finally, the conceptualisation of patient safety and of how it is best preserved can be compared between the different professional groups involved in the study (medical, nursing, and allied health staff). Much research in this area to date has focussed on the perceptions and practices of single occupational groups (especially nurses).

### Data collection phases

Atkinson et al. [30] emphasise the importance in ethnography of systematic analysis of multiple cultural forms, including narratives, visual representations, discourse, material culture, and space. The data collection framework illustrated in Table 1 aims at enabling such a rounded analysis. The different observation modes proposed in phases 1-4 are also inspired by Strauss' methodological descriptions of his ethnographic study of psychiatric hospitals in the 1960s [31]. Czarniawska's more recent recommendations on innovative ethnography in modern societies have further influenced the framework; she argues that 'traditional ethnography is not enough anymore' [[32]: 7], because of the multiplicity of times, places and modes of communication in which contemporary professionals accomplish their work, and because of the more focussed interests of organisational ethnographers who wish to analyse the production of some phenomenon (such as patient safety) rather than the operations of an entire group or society. The methods of data collection will centre on structured and unstructured unobtrusive observations of staff and setting. These observations will be supplemented by field interviews (informal interviews during the course of observation) and by more formal interviews after the conclusion of the observation phases of the study. A final phase comprising a social network questionnaire will be undertaken in both settings.

The field researcher (JP) will spend a maximum of 16 hours per week, for 10 weeks, in each of the two settings. This is a maximum time limit because at each phase, observations will continue until thematic saturation has been reached and no new findings are emerging. This time includes the observational and interview phases of the study (phases 1-5). The social network questionnaire will be administered to staff in both settings after the observational and interview data collection phases have been completed.

### Observation phases

The main aim of ethnography is to build up a picture of the cultural and social system under study through extended researcher exposure to the setting and the building of a relationship of trust with participants [33]. Observation of what people actually do provides a useful comparison to data acquired through methods which only capture what people *say *they do (such as interviews). It does not rely on participants' memories, and goes some way to overcoming the problem of people describing their jobs in an abstract way that is 'expected' of them. The researcher can choose to play a particular role during observations, ranging from full participation and membership in the setting, to complete observer, having no interaction with participants [34].

In this study, the field researcher will employ what Adler and Adler term the 'peripheral-member-researcher' role, where the identity of the researcher is clearly maintained, where observations are largely unobtrusive, but where interaction with participants and participation in some non-clinical tasks (such as making coffee, helping to set up a room for an activity) is undertaken. Opportunistic field interviews will be used as needed to clarify understandings and elucidate more detail about individuals' viewpoints. This period will also be used to collect relevant documents for later analysis.

Field notes will be taken as soon as possible after (or if appropriate during) observations, with at least two days per week dedicated to writing them up in full. This will allow concurrent analysis of emerging themes which act to structure more focussed observations as the research progresses [35]. As wide a range of day time shifts as possible will be observed. Where consent is given, audio recordings will be made of interviews (phase 5), and of any staff meetings observed in phases 1-4.

#### Phase 1: Initial unstructured observations

Spradley [36] described the process of ethnographic observation as a funnel, in that initial observations are relatively unfocussed, designed to 'get a feel for the setting' and to begin to build rapport with its members. As time goes on and patterns or themes start to emerge from the data, observations become more focussed on particular people, events, and places. The initial (unstructured) phase will be used here for unobtrusive observations of patterns of activity, staff roles, layout of the settings, and to identify key informants and key locations and practices for phases 2, 3 and 4 of the study (see table 1 for details of the phases).

#### Phase 2: Shadowing key informants

Shadowing has been used as an observational technique by ethnographers interested in mapping how professional practice and organising are accomplished across time and space [32]. This is an especially useful technique to gain an insight into the everyday working lives of different professionals and how they interact with other professionals and with the environment to achieve their tasks. Depending on the role, seniority, and experience in the setting of the person being shadowed, this is also an opportunity to observe inter-professional communication, power dynamics and the socialisation of newcomers into the setting and its 'norms' of safe practice.

Between six and eight key informants will be shadowed in each setting. These informants will be chosen to be representative of the professional groups employed at the setting. The researcher will accompany the key informant as they go about their work, and each informant will be shadowed for a maximum of eight hours, which may be one entire shift or be spread over several shifts as circumstances allow.

#### Phase 3: Stationary observations in key locations

The third phase will entail the researcher remaining *in situ *for a total period of 8 hours in each of 2 locations which have been identified in the previous phase, such as the staff office or nurses' station. The purpose of stationary observations is to listen to informal conversations in places where staff gather together, to view the patterns of their movement, use of space, and the role of key artefacts in the constitution of the phenomena of interest. The value of the observation of materiality and space in the revelation of aspects of the accomplishment of practice and meaning which are not revealed in text or talk is increasingly recognised by ethnographers using a practice approach [e.g. [37,38]]. Such approaches emphasise the 'interdependency of the human and the material' [[39]: 310].

#### Phase 4: Tracing key practices

Nicolini [22], a practice theorist, describes an innovative method of observation where, rather than shadowing people, he followed the conduct of particular practices which he had previously identified as key to the phenomenon he was studying (telemonitoring of cardiac patients). He does this by, for example, following the people, artefacts and documents associated with a particular practice, such as attending meetings on the subject, visiting other sites of the practice, observing nurses telephoning patients to do the monitoring, and analysing the charts they used to keep track of the remote monitoring process. By doing this he was able to assess the micro-, meso- and macro-level factors influencing and being influenced by the practice of telemedicine.

In the present study, two practices specifically related to safety preservation or to assessment of a near miss or incident will be followed for a maximum of one shift (eight hours) each. This will enable the mapping of factors contributing to, shaping, and being shaped by, the practices under study, providing a detailed picture of how safety is enacted. The artefacts, people, tasks and discourses employed in accomplishing the practice will be noted.

### Interview phase

#### Phase 5: Interviewing key informants

The interview is used in ethnography for two principal reasons: to gain information about the topic of interest, and to garner samples of participants' discourse and narrative which can be used to study how people construe and construct their reality, how they order their experience, the resources they use to make meaning, and so on [33]. In addition, this research will use the interview phase to check participants' reactions to emerging findings from the observational phases. Such 'member checking' is a recognised way of validating findings but can also stimulate further discussion in an attempt to uncover more about how participants understand their world [40].

Between six and eight key informants will be interviewed in each of the two settings. These will be the same informants who participated in the shadowing phase (Phase 2). The types of questions asked will be determined by the earlier observation phases, because ethnographic interviewing employs the language and concepts used by participants rather than the concepts of social science [41]. Ethnographic interviewing uses open-ended questions, and the exact order and wording of questions is not pre-determined, although the interviewer goes in with a list of issues to be covered [33]. Questions proceed reflexively in response to the interviewee's answers, whilst steering the conversation back to the issues of interest.

Information collected during these interviews will depend to a large extent on findings during the earlier observation phases of the study, but in broad terms will comprise the following:

a. Information about how staff keep things running smoothly in their service, intended to get staff to reflect on and make explicit the usually taken-for-granted assumptions, norms and rules according to which they accomplish safe care.

b. Information about perceived barriers to and enablers of a smoothly-running service.

c. Information derived from participants' reflections on the researcher's emerging findings from the previous (observation) phases of the study.

### Social network analysis phase

#### Phase 6: Safety communication network analysis

To complement the observational fieldwork and interviews, the settings under consideration will be studied using social network analysis (SNA). SNA involves the mapping of ties or relationships between members of a selected group of people and the analysis of the structure of the network [42]. The idea of SNA is to show how social structure impacts on behaviour or other variables of interest. In the context of this study, SNA will be used to help compensate for some of the limitations of observation techniques (where the researcher can only see and record a small sub-set of interactions) and enable an overall picture of relations on the ward or team to be built. This will also help determine how typical of the setting observed interactions are. Being a highly structuralist approach to analysing human relations, the SNA will provide a useful methodological contrast to the 'bottom up' understandings garnered from the observation phases.

A social network questionnaire will be devised which will ask all members of the two study settings about with whom they interact on issues of safety and risk, and how often. In keeping with the emergent and exploratory nature of ethnographic research, the wording and structure of the questionnaire will be informed by the observation and interview phases of the study. Such a questionnaire can be used to map patterns of interaction, to reveal whether such interaction occurs mostly between members of the same professional group, same gender, level of experience, or other variable of interest. Interactions with staff outside of the two settings will also be taken into account. The nature of communications between clinical staff and clinical governance or risk management staff, for example, will provide valuable data, especially in the context of formal mechanisms for reporting and monitoring safety incidents. The contents of the questionnaire will be devised based on findings in earlier phases of the study, as its principal purpose is to provide a point of comparison or validation of the earlier findings.

Mapping connections between different settings may help shed light on important safety issues which affect the quality of patient care, such as continuity of care between settings and discharge practice. Analysis of the social networks present in the mental health settings under study might help to show whether organisational and professional structures and cultures help or hinder learning and practice around patient safety. Close examination of interaction on issues of patient safety might reveal how structural factors can constrain or enable practitioners' efforts to avoid problems and incidents and learn from them when they do occur.

The social network questionnaire will be designed so that it takes each staff member less than 30 minutes to complete. Its questions will ask staff members who they interact with on issues of safety, how frequently, and the nature of the interactions.

### Data analysis

Ethnography is by its nature an inductive methodology. As findings emerge or phenomena of interest to the research questions emerge, methods of data collection and analysis may evolve or change [12,33].

In light of this, the process of analysis will be undertaken alongside data collection. Texts on qualitative data analysis generally recommend such an approach, where findings of ongoing analysis serve to help focus future data collection. Miles and Huberman [[43]: 50] encourage researchers to 'cycle back and forth between thinking about the existing data and generating strategies for collecting new, often better data.' To aid in the reflexive monitoring of progress and direction, a research journal will be kept in which emerging analytic ideas and hypotheses will be noted, developed, and fed back into the data collection process.

As data from multiple sources will be collected during the different stages of the study, multiple methods of analysis will be needed in order to understand what the data is saying about how staff understand safety and the implications these understandings have for practice. Although the overarching analytical approach will be theoretically informed by actor-network theory, a type of analytical 'toolbox', advocated by Nicolini [22], will be employed, where different practice approaches will be used in tandem as they best illuminate the data to be analysed. For example, use may be made of the empirical approaches employed by structuration theorists [following [44]], activity theorists [following [45]], nexus analysts [46], and ethnomethodologists [following [47]]. A detailed and integrated analytic framework informed by these practice theories will be developed once familiarity with the setting is obtained, and following ongoing engagement with these theories themselves.

As the research questions outlined above imply, this study will set out to analyse both *how *understandings of safety are developed and reproduced by mental health professionals (through interaction and learning), and *what *these understandings consist of for different people and in different contexts, as recommended by Silverman [48].

The data analysis process will employ the following techniques:

a. Iterative coding of the written data (from fieldnotes, interview and meeting transcripts, and existing documentation) in order to determine patterns of meaning using thematic, narrative and semiotic analysis.

b. Use of software aids such as NVivo to help with coding and organising textual data, and Leximancer, an automated data mining software, to obtain an overview of conceptual relationships present in the texts.

c. Validation of interpretations: methodological triangulation of findings across observation, interviews and social network analysis; member checking.

### Ethics approval

Ethics approval has been granted by the relevant Area Health Service HREC and University of New South Wales HREC 10384.

## Discussion

There are multiple theoretical, methodological, policy and practice contributions of this research. Theoretically, the use of practice theory and actor-network ideas as sensitising concepts for studying the conceptual and material constitution of patient safety is an innovative approach. It is an approach which contrasts with (and complements) the theoretical orthodoxy of much existing patient safety research by approaching safety not from the abstract and instrumentalist system level but from the level of professional practice. This enables a detailed examination of the respective roles of different safety imperatives (emanating from history or policy, for example) as well as the role of the social and material worlds in the constitution of safety as it unfolds.

Methodologically, the study of patient safety in the context of multidisciplinary care contrasts with much existing ethnographic work in mental healthcare, which tends to focus on the experiences of one professional group in isolation. The comparison of conceptualisations of safe care between professional groups, as well as between community and inpatient settings, is another unique contribution of the research, as much research into safety in mental healthcare takes place in the inpatient setting. The data collection framework will also enable comparison of findings derived from tracing how actor-networks are built by the actors themselves ('insider' view), against the more structuralist or 'outsider' approaches of traditional social network analysis [49].

In policy terms, this study is designed to demonstrate the importance of taking into account professionals' conceptualisations of safety and their strategies for accomplishing it when designing locally relevant safety improvement initiatives. It will be an opportunity for an assessment of the relative importance of formal policy as one of the many elements driving the safety agenda at the level of local services. It is also hoped that the findings will enable a re-examination of the theoretical orthodoxy of the patient safety movement. In terms of clinical practice, the findings of this study will provide an opportunity for stakeholders in mental health to reflect on the relative contribution of the patient, the nature of the mental health setting and care regimes in the preservation of safety. Uncovering the taken-for-granted assumptions which underlie professionals' decisions in relation to safety and risk is one way in which such reflection could guide local improvements in patient safety.

## Conclusion

Two advocates of the ethnographic approach to patient safety research eloquently encapsulate the rationale behind the present study. Bosk [[16]: 1-2], a pioneer of the ethnographic approach to safety in healthcare, argues that we may be asking the wrong questions, or too narrow a set of questions, when it comes to patient safety research. Nearly thirty years after his seminal study of surgeons' conceptualisations of error [50], he finds that patient safety research is still almost exclusively asking 'How might adverse medical events be prevented?' He advocates that we turn to different questions, such as 

'...how do workers in a medical setting define what is an error? How do they understand what causes error? And how do they respond to errors? ... this second view...concentrates on the negotiation of the meaning of the term error on the 'shop floor'... [their] meanings are not fixed but are fluid and flexible, highly dependent on context.'

Interest in using qualitative approaches to understand uncertainty and mistakes in medicine waned after the heyday of Bosk [50], Millman [51] and Paget [52], with little work emerging in this vein until the latter half of the 2000s. This resurgence coincided with the abovementioned realisation that improvement efforts centred on human factors engineering and techniques imported from 'high reliability' industries such as aviation and nuclear power were having only modest impacts. The 'second wave' of interest in ethnographic approaches to understanding patient safety is concerned with better understanding the role of safety in the everyday world of clinicians. Dixon-Woods [[13]: 11-12] in an article entitled *'Why is patient safety so hard?*' further elaborates the potential contribution of this methodological approach:

'[There is a]...need for solutions to be based on a sound understanding of the nature of the problems and what kinds of approaches are likely to be best suited to resolving them. Such an understanding requires insight into the complexities of the networks in which hospital workers are embedded, and of how alternative conceptions of what is 'safe' or 'good practice' may prevail, conditioned by coping with competing priorities, clinical uncertainties, organisational pressures, resource inadequacies, and efforts at professional boundary maintenance.'

It is this nuanced understanding of the complex of elements coalescing to enable or constrain the performance of safe care that we seek to further in this study. Its focus on tracing connections between the heterogeneous elements of mental health practice will enable the articulation of safety as an actively constituted, continuously emergent performance. This will involve turning away from the current focus on tracing the trajectories of adverse events, towards tracing the network of connections enabling safety to be maintained. Such an approach heeds a recent call [11] for social scientists to contribute new ways of studying and improving patient safety rather than standing on the sidelines of the debate as critics.

## Competing interests

This research is supported under the Australian Research Council's Discovery Projects funding scheme [project number DP0986493]. There are no financial or non-financial competing interests.

## Authors' contributions

JP is the primary author of this manuscript, and is responsible for the conceptual and methodological development of the described project which forms the basis of her PhD research. JB, JT and PN contributed to the conceptual and methodological refinement of the project; they also performed critical appraisal and editing of this manuscript. All authors read and approved the final manuscript.

## Pre-publication history

The pre-publication history for this paper can be accessed here:

http://www.biomedcentral.com/1472-6963/11/100/prepub
